# An improved grey wolf optimization algorithm for 3-D UWB indoor positioning

**DOI:** 10.1371/journal.pone.0352111

**Published:** 2026-06-18

**Authors:** Jingmei Zhou, Bing Li, Shanshan Yang, Chungang Liu

**Affiliations:** 1 Hebei Normal University, Vocational and Technical School, Shijiazhuang, Hebei, China; 2 Hebei Provincial Innovation Center for Wireless Sensor Network Data Application Technology, Shijiazhuang, Hebei, China; 3 Hebei Provincial Key Laboratory of Information Fusion and Intelligent Control, Shijiazhuang, Hebei, China; 4 Department of Information Engineering, Hebei University of Environmental Engineering, Qinhuangdao, Hebei, China; University of New England School of Science and Technology, AUSTRALIA

## Abstract

Traditional Ultra-wideband (UWB) positioning algorithms cannot achieve ideal positioning results when facing multipath effects and non-line-of-sight (NLOS) factors. In order to improve the accuracy of UWB indoor positioning, this paper proposes an improved grey wolf optimization (GWO) algorithm for 3-D UWB indoor positioning in the NLOS environment. First, the Chan algorithm is used for initial tag positioning. Then, the search area of the GWO algorithm is constructed with the initial positioning result as the center. Next, the GWO algorithm’s optimization accuracy and convergence speed are improved through the improvement of Tent chaotic mapping, nonlinear convergence factor based on cosine function, and dynamic inertia weight factor. Finally, the optimized position of the tag is determined using the improved GWO algorithm. Experimental results show that this algorithm can converge to the global optimal solution faster and achieve higher positioning accuracy in complex experimental environments. Compared with the Chan, ChanTaylor, particle swarm optimization (PSO), GWO, and enhanced grey wolf optimization (AGWO) algorithms, the average positioning accuracy of the proposed algorithm is improved by 62.92%, 66.43%, 45.71%, 40.91%, and 37.76% respectively, demonstrating its high practical value.

## 1 Introduction

With the rapid development of wireless communication technology and the Internet of Things, services based on indoor positioning technology are gradually becoming the focus of attention. Among them, Ultra-Wideband (UWB) technology [1] has excellent anti-multipath effects, flexible data transfer rates, and high-ranking accuracy, making it widely used in intelligent warehousing, autonomous driving, and other fields [2].

Compared with traditional positioning methods such as the Chan and Taylor algorithms [3], the intelligent optimization algorithm has a better positioning effect, especially in complex environments with a significant influence of multi-path and non- line-of-sight (NLOS) [4], and can effectively improve positioning accuracy. For example, particle swarm optimization (PSO) [5], sine cosine algorithm (SCA) [6], genetic algorithm (GA) [7], firefly algorithm (FA) [8] and water cycle algorithm (WCA) [9]. These algorithms treat the indoor positioning problem as an optimization problem [10], which can reduce the computational complexity and improve the indoor positioning accuracy. In recent years, an increasing number of researchers have combined indoor positioning technology with optimization algorithms. Cao*et al*. [11] improved the position estimation accuracy of UWB positioning systems in complex environments by combining the variational Bayesian unscented Kalman filter (VBUKF), total least square (TLS), and WCA optimization algorithm. The TLS positioning results were optimized using the WCA optimization algorithm. However, the WCA optimization algorithm has issues, such as falling into a local optimum, which can affect the optimization results of positioning. Lakshmi *et al*. [12] proposed a hybrid PSO positioning method based on an improved Chan algorithm. This method utilizes ensemble learning particle swarm optimization (ELPSO) to optimize the tag positioning results and improve the accuracy in both 2-D and 3-D environments. However, this method does not impose any restrictions on the search space of the PSO algorithm, resulting in an extensive search range and consequently affecting the convergence speed of the algorithm. Yang *et al*. [13] proposed a 3-D UWB indoor positioning method based on the improved PSO algorithm. According to the improved PSO algorithm, the optimal parameters of the Kalman filter (KF) were determined to correct the tag position, thus improving the positioning accuracy. However, this method has the problem of falling into the local optimum, and the convergence speed is slow. Xian *et al*. [14] proposed a positioning method based on fuzzy logic and Gauss-Cauchy cuckoo search (ICS-FG) to minimize the positioning error of wireless sensor networks. The proposed Gauss-Cauchy strategy significantly improved the search accuracy of the algorithm. However, this method also has the problem of slow convergence. Liu *et al*. [15] proposed a positioning method based on the improved whale optimization algorithm (IWOA). Firstly, the Min-Max algorithm was used to obtain the search area of IWOA. Then, IWOA is used to find the optimized position of the target node within the search area. This method can reduce positioning errors. However, IWOA still has the problem of falling into local optimum, and the optimization accuracy needs further improvement. Ghorpade *et al*. [16] proposed a novel grey wolf optimization (GWO) technique for node positioning, which can significantly reduce positioning errors. The GWO algorithm has the advantages of simple structure and short optimization time, but there are problems, such as falling into local optimum [17]. In addition, intelligent optimization algorithms often adopt a global search strategy, which makes it challenging to find the optimum solution in indoor areas due to the large search area. This situation leads to a slower convergence speed of the algorithm and greatly wastes computing resources [18].

To better apply the GWO algorithm to the field of indoor positioning and improve accuracy in the NLOS environment, this paper proposes an improved GWO algorithm for 3D UWB indoor positioning. Our previous work [19] has already introduced improved Tent chaotic mapping and a nonlinear convergence factor based on cosine function to enhance the performance of optimization algorithms. Building upon this foundation, this paper further proposes a dynamic inertia weight position update mechanism and integraand deeply integrates three strategies: the improved Tent chaotic mapping, the upgraded cosine-based nonlinear convergence factor, and the dynamic inertia weight, and develops a Multi-Strategy Grey Wolf Optimization (MSGWO) algorithm specifically tailored for 3D UWB indoor positioning. The main contributions of this paper are as follows:

1) A Kalman filter algorithm is first introduced to preprocess TDOA measurement values, effectively reducing the impact of multipath effects and NLOS errors before positioning calculations.
2) The Chan algorithm is then used for initial tag positioning based on the filtered TDOA data, and the search area of the GWO algorithm is constrained based on the initial positioning results.
3) To address the issue of the GWO algorithm easily falling into local optima and slow convergence, a multi-strategy improved grey wolf optimization (MSGWO) method is proposed. This method integrates three strategies: improved Tent chaotic mapping for population initialization, a nonlinear convergence factor based on cosine function, and a novel adaptive inertia weight factor introduced in this study.
4) This paper proposes a complete 3D UWB indoor positioning framework that sequentially applies Kalman-filtered TDOA measurements, Chan-based initial positioning, and MSGWO optimization to achieve higher positioning accuracy and faster convergence in NLOS environments.

## 2 Materials and methods

### 2.1 Positioning model

#### 2.1.1 TDOA positioning principle.

**Fig 1:**
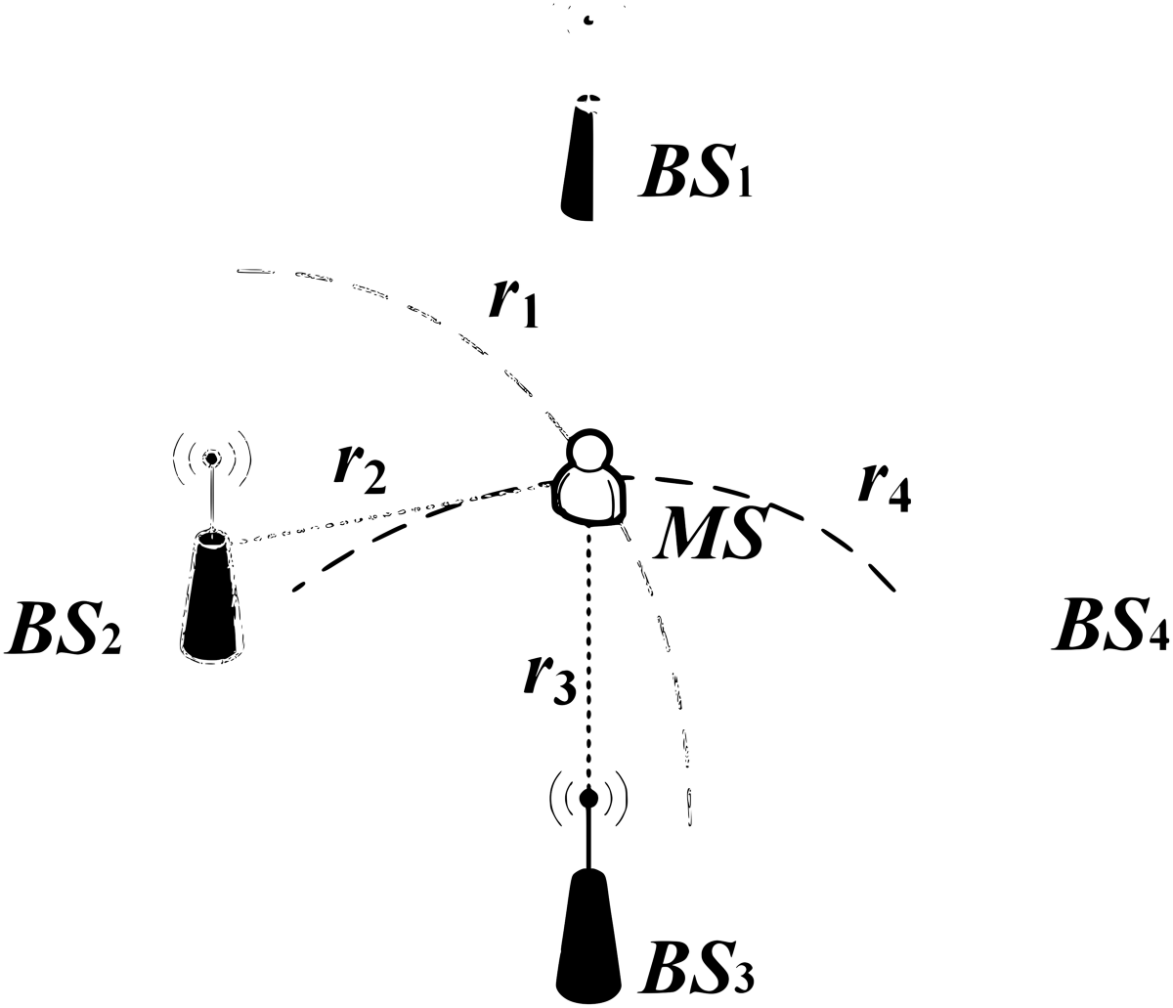
TDOA positioning principle. The mobile station (MS) emits signals received by four base stations ($BS_{1}-BS_{4}$). Time differences of arrival (TDOA) are converted to range differences ($r_{1}-r_{4}$), forming hyperbolic equations that are solved to determine the 3D coordinates of the MS.This forms the initial positioning basis for the proposed MSGWO algorithm.

The principle of time difference of arrival (TDOA) positioning is to measure the time difference of signal arrival between multiple base stations and then use these time differences to calculate the position of the tag [20,21]. The TDOA positioning principle is shown in Fig 1.

Consider a scenario where m base stations with known positions are used to locate the position of a tag in a 3D space. Let the coordinates of the base stations be denoted by $BS_{i}(x_{i},y_{i},z_{i}),i=1,2,...,m$, and the tag coordinate be denoted by *MS* (*x*, *y*, *z*).

The true distance from the tag to the *i*-th base station is:


$$\begin{array}{c} r_{i}^{0}=\sqrt{{\left(x_{i}-x\right)}^{2}+{\left(y_{i}-y\right)}^{2}+{\left(z_{i}-z\right)}^{2}}=ct_{i}^{0} \end{array}$$ (1)

where *t* is the propagation speed of electromagnetic wave signals,$c=3\times 10^{8}\;m/s$ and $t_{i}^{0}$ represents the time when the *i*-th base station receives the signal emitted by the tag.

The time difference between the arrival of the tag at base station *BS*1 and base station *BSi* is as follows:


$$\begin{array}{c} t_{i,1}^{0}=t_{i}^{0}-t_{1}^{0} \end{array}$$ (2)

The distance difference between the arrival of the tag at base station *BS*1 and base station *BSi* is as follows:


$$\begin{array}{c} r_{i,1}^{0}=ct_{i,1}^{0}=r_{i}^{0}-r_{1}^{0} \end{array}$$ (3)

Due to the complexity of the indoor environment, the actual TDOA measurement values are as follows:


$$\begin{array}{c} r_{i,1}=r_{i}^{0}-r_{1}^{0}+n_{i,1} \end{array}$$ (4)

where $n_{i,1}$ represents the TDOA measurement noise, generally, $n_{i,1}$ follows the Gaussian distribution with zero mean and standard deviation σ, denoted as $n_{i,1}\sim (0,\sigma^{2})$.

#### 2.1.2 Kalman filter.

Kalman Filter [22] is a recursive optimal estimation algorithm based on the state equation of linear systems. By fusing the “a priori state information” of the system and “real-time observation data,” it dynamically suppresses noise interference and outputs high-precision state estimation values, which is suitable for state prediction and correction of dynamic systems under Gaussian noise. The mathematical model of the algorithm is as follows:

State equation


$$\begin{array}{c} x_{t}=Ax_{t-1}+W_{t-1} \end{array}$$ (5)

Observation equation


$$\begin{array}{c} z_{t}=Hx_{t}+V_{t} \end{array}$$ (6)

Where: *A* denotes the state transition matrix; $X_{t}$ represents the state quantity of the system at time *t*; $W_{t-1}$ is the process noise; $Z_{t}$ stands for the observa*t*ion quantity at time *t*; *H* is the observation matrix; and $V_{t}$ denotes the observation noise. The implementation process of the KF is detailed as follows:

State Prediction


$$\begin{array}{c} {\hat{X}}_{t}^{-}=A{\hat{X}}_{t-1} \end{array}$$ (7)

Prediction Covariance Matrix


$$\begin{array}{c} P_{t}^{-}=AP_{t-1}A^{T}+Q \end{array}$$ (8)

Kalman Gain Matrix


$$\begin{array}{c} K_{t}=P_{t}^{-}H^{T}(HP_{t}^{-}H^{T}+R{)}^{-1} \end{array}$$ (9)

State Update at Time


$$\begin{array}{c} {\hat{X}}_{t}={\hat{X}}_{t}^{-}+K_{t}(Z_{t}-H{\hat{X}}_{t}^{-}) \end{array}$$ (10)

Covariance Matrix Update


$$\begin{array}{c} P_{t}=(I-K_{t}H)P_{t}^{-} \end{array}$$ (11)

Where: ${\hat{X}}_{t-1}^{+}$ is the optimal estimation value of the state at time t−1; $P_{t-1}^{+}$ represents the covariance matrix between the true filtered value and the optimal estimation value at time t−1; *Q* denotes the system noise covariance; *R* is the observation noise covariance; and *I* stands for the identity matrix.

#### 2.1.3 Chan algorithm.

The Chan algorithm [23] is a positioning method based on TDOA. It determines the position of the tag by solving the system of nonlinear hyperbolic equations and applies weighted least squares (WLS) [24] multiple times to correct the positioning error. The mathematical model of the Chan algorithm is as follows: Let the position of

Let the position of the base stations be $BS_{i}(x_{i},y_{i},z_{i}),i=1,2,...,m$, the tag position be *MS*(*x*,*y*,*z*), and the distance between the tag and the base stations be $r_{i}$.


$$\begin{array}{c} r_{i}=\sqrt{{\left(x-x_{i}\right)}^{2}+{\left(y-y_{i}\right)}^{2}+{\left(z-z_{i}\right)}^{2}} \end{array}$$ (12)

$$\begin{array}{c} r_{i}^{2}={\left(r_{i,1}+r_{1}\right)}^{2}=r_{i,1}^{2}+2r_{i,1}r_{1}+r_{1}^{2} \end{array}$$ (13)

According to Eq 12 and 13, it can be concluded that:


$$\begin{array}{c} r_{i,1}^{2}+2r_{i,1}r_{1}=K_{i}-K_{1}-2xx_{i,1}-2yy_{i,1}-2zz_{i,1} \end{array}$$ (14)

where $r_{i,1}=r_{i}-r_{1}$; $x_{i,1}=x_{i}-x_{1}$; $y_{i,1}=y_{i}-y_{1}$; $z_{i,1}=z_{i}-z_{1}$; $K_{i}=x_{i}^{2}+y_{i}^{2}+z_{i}^{2}$.

By organizing Eq 14, it can be concluded that:


$$\begin{array}{c} \frac{1}{2}\left(r_{i,1}^{2}-K_{i}+K_{1}\right)=-\left[\begin{array}{cccc} x_{i,1} & y_{i,1} & z_{i,1} & r_{i,1} \end{array}\right]\left[\begin{array}{c} x\\ y\\ z\\ r_{1} \end{array}\right] \end{array}$$ (15)

Let $z_{a}=[z_{p}^{T}\;r_{1}{]}^{T}$ be the unknown variable, where $z_{p}=[x\;y\;z{]}^{T}$. Establish a linear system of equations with the presence of TDOA measurement noise:


$$\begin{array}{c} e=h-G_{a}z_{a} \end{array}$$ (16)

where $h=\frac{1}{2}[\begin{array}{c} r_{2,1}^{2}-K_{2}+K_{1}\\ r_{3,1}^{2}-K_{3}+K_{1}\\ \vdots \\ r_{m,1}^{2}-K_{m}+K_{1} \end{array}];G_{a}=[\begin{array}{cccc} x_{2,1} & y_{2,1} & z_{2,1} & r_{2,1}\\ x_{3,1} & y_{3,1} & z_{3,1} & r_{3,1}\\ \vdots & \vdots & \vdots & \vdots \\ x_{m,1} & y_{m,1} & z_{m,1} & r_{m,1} \end{array}].$

Assuming that the elements in $z_{a}$ are independent of each other, perform the first WLS on Eq 16 to obtain the first estimated position of the tag:


$$\begin{array}{c} z_{a}=\text{arg}\min\left\{(h-G_{a}z_{a}{)}^{T}\Psi^{-1}(h-G_{a}z_{a})\right\}=(G_{a}^{T}\Psi^{-1}G_{a}{)}^{-1}G_{a}^{T}\Psi^{-1}h \end{array}$$ (17)

Where Ψ is the covariance matrix of error vector *e*, $\Psi =E[ee^{T}]=c^{2}BQB$, $B=diag\{r_{2}^{0},r_{3}^{0},...,r_{m}^{0}\}$, $r_{i}^{0}$ is the true distance between the tag and the *i*-th base station, *Q* is the covariance matrix of TDOA and $Q=diag\{\sigma_{2,1}^{0},\sigma_{3,1}^{0},...,\sigma_{m,1}^{0}\}$.

To obtain a more accurate position of the tag, further optimization of the initial estimation result is required to establish a second estimation linear equation:


$$\begin{array}{c} \tilde{e}=\tilde{h}-{\tilde{G}}_{a}{\tilde{z}}_{a} \end{array}$$ (18)

where $\tilde{h}=[\begin{array}{c} (z_{a,1}-x_{1}{)}^{2}\\ (z_{a,2}-y_{1}{)}^{2}\\ (z_{a,3}-z_{1}{)}^{2}\\ z_{a,4}^{2} \end{array}];{\tilde{G}}_{a}=[\begin{array}{ccc} 1 & 0 & 0\\ 0 & 1 & 0\\ 0 & 0 & 1\\ 1 & 1 & 1 \end{array}];{\tilde{z}}_{a}=[\begin{array}{c} (x-x_{1}{)}^{2}\\ (y-y_{1}{)}^{2}\\ (z-z_{1}{)}^{2} \end{array}].$

The covariance matrix of $\tilde{e}$ is as follows:


$$\begin{array}{c} \tilde{\Psi }=E\left[\tilde{e}(\tilde{e}{)}^{T}\right]=4\tilde{B}Cov\left[z_{a}\right]\tilde{B} \end{array}$$ (19)

where $\tilde{B}=diag\{x^{0}-x_{1},y^{0}-y_{1},z^{0}-z_{1},r_{1}^{0}\}$; $Cov[z_{a}]=E[\Delta z_{a}\Delta z_{a}^{T}]=(G_{a}^{T}\Psi^{-1}G_{a}{)}^{-1}$; $\Delta z_{a}$ is the estimation error of $z_{a}$.

Perform the second WLS on Eq 18 to get the second estimated position of the tag:


$$\begin{array}{c} {\tilde{z}}_{a}={\left[({\tilde{G}}_{a}{)}^{T}(\tilde{\Psi }{)}^{-1}{\tilde{G}}_{a}\right]}^{-1}\left[({\tilde{G}}_{a}{)}^{T}(\tilde{\Psi }{)}^{-1}\tilde{h}\right] \end{array}$$ (20)

The position of the tag calculated by the Chan algorithm is as follows:


$$\begin{array}{c} z_{p}=\pm \sqrt{{\tilde{z}}_{a}}+[\begin{array}{ccc} x_{1} & y_{1} & z_{1} \end{array}{]}^{T} \end{array}$$ (21)

#### 2.1.4 Traditional grey wolf optimization algorithm.

The GWO algorithm is an optimization algorithm based on the collective behavior of grey wolf populations, proposed by Mirjalili *et al*. in 2014 [25]. This algorithm simulates the cooperation and competition relationship between the leader and members of the grey wolf pack, searching for the optimal solution by exploring the positions of individuals in the search space.

The GWO algorithm hierarchically divides the grey wolf pack and assigns different responsibilities to wolves at each level to complete various tasks during the hunting process. The highest-level wolf is called α followed by β, δ, and *w*. The α wolf is the leader of the entire group, and all members must obey its commands. The β wolf serves as the assistant to α, assisting in managing the work. Theδ wolf follows the leadership of α and β wolves, responsible for surveillance and defending the entire group’s safety. The *w* wolf is positioned at the bottom of the pyramid and is responsible for completing tasks given by wolves from higher levels.

The GWO algorithm consists of three steps: surrounding, hunting, and attacking.

**Fig 2:**
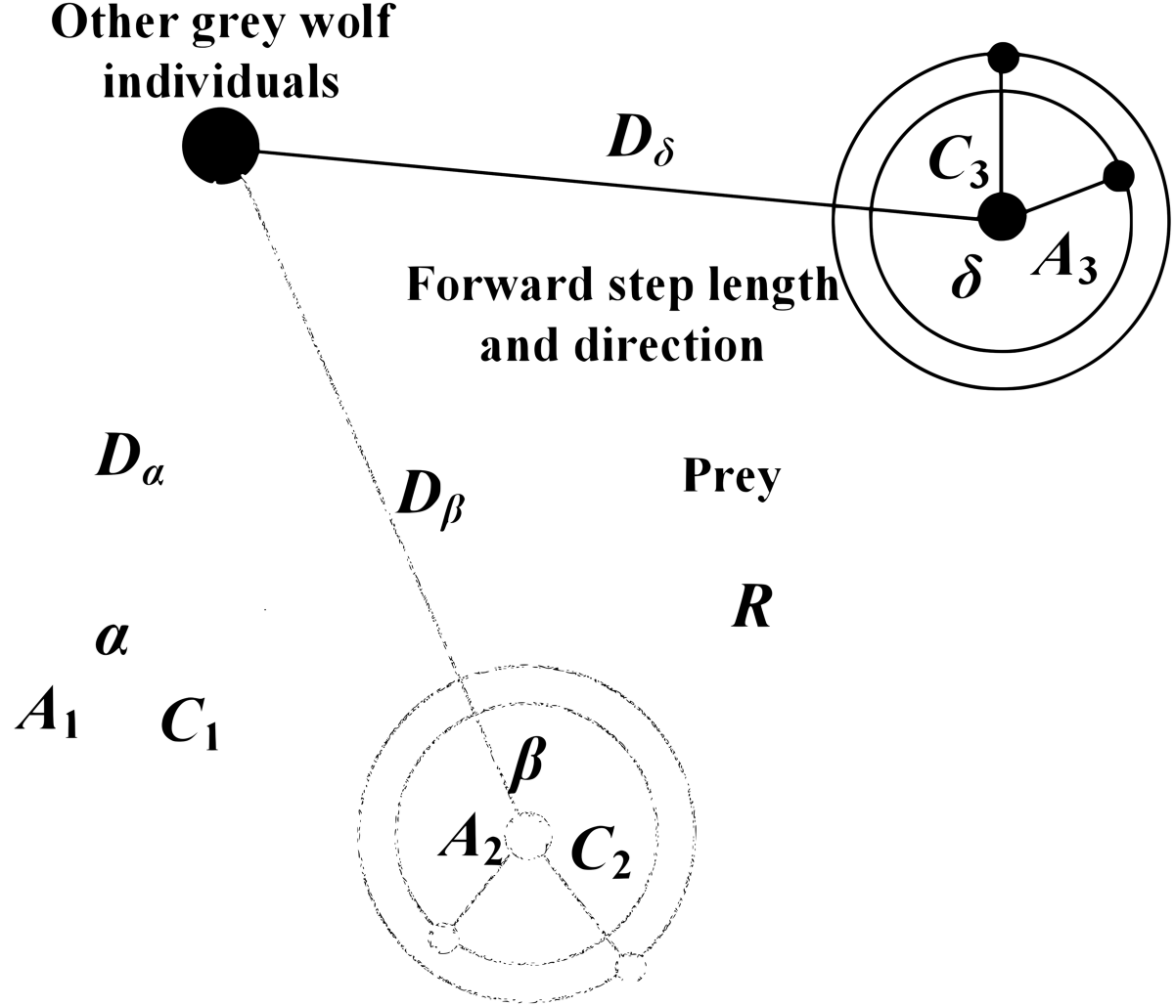
Hunting process of grey wolves. The process is guided by the dominant wolves (α, β, and δ, shown in blue, green, and purple), which estimate the prey’s position and direct the pack. Other grey wolf individuals update their positions based on these leaders, adjusting both the forward step length and direction to approach the prey. This mechanism balances global exploration and local exploitation: dispersed search occurs when |*A*| > 1, while concentrated local search occurs when |A|≤1.

1) Surrounding prey. When wolves search for prey, they gradually approach and surround it. The mathematical model is as follows:
  $$\begin{array}{c} D=\left|CX_{p}(t)-X(t)\right| \end{array}$$ (22)
  $$\begin{array}{c} X(t+1)=X_{p}-A\times D \end{array}$$ (23)
  $$\begin{array}{c} A=2ar_{1}-a \end{array}$$ (24)
  $$\begin{array}{c} C=2r_{2} \end{array}$$ (25)
  $$\begin{array}{c} a=2-2\times \frac{t}{t_{\max}} \end{array}$$ (26)
  where $X_{p}$ represents the prey position; *X*(*t*) represents the grey wolf individual’s position; *D* represen*t*s the distance between the grey wolf individual and the prey; *A* and *C* are coefficient vectors, with *A* ranging from [−2, 2] and *C* ranging from [0, 2]; *r*_1_ and *r*_2_ are random numbers within the range of [0, 1]; *a* is a linear convergence factor decreasing from 2 to 0; *t* represents the current i*t*eration number; $t_{max}$ represents the maximum number of iterations.
2) Hunting. The α, β, and δ wolves make rough judgments on the position of prey and then guide the entire wolf pack, allowing the other grey wolves to be aware of the prey’s position. The position of the *w* wolf is updated based on the α, β, and δ wolves. The hunting process of the grey wolves is shown in Fig 2. The mathematical model is as follows:
  $$\begin{array}{c} \left\{ \begin{array}{c} D_{\alpha }=\left|C_{1}\times X_{\alpha }(t)-X(t)\right|\\ D_{\beta }=\left|C_{2}\times X_{\beta }(t)-X(t)\right| \end{array}\right. \end{array}$$ (27)
  $$\begin{array}{c} \left\{ \begin{array}{c} X_{1}(t+1)=X_{\alpha }(t)-A_{1}\times D_{\alpha }\\ X_{2}(t+1)=X_{\beta }(t)-A_{2}\times D_{\beta } \end{array}\right. \end{array}$$ (28)
  $$\begin{array}{c} X(t+1)=\frac{X_{1}(t+1)+X_{2}(t+1)+X_{3}(t+1)}{3} \end{array}$$ (29)
  where $D_{\alpha }$, $D_{\beta }$, $D_{\delta }$ and represent the distances between other individual grey wolves and the α,β, and δ wolves, respectively; $X_{\alpha }(t),X_{\beta }(t),X_{\delta }(t)$ and represent the positions of the α, β*, and* δ wolves in the *t*-th iteration, respectively.
3) Attack behavior. When the wolf pack knows the position of the prey, the wolf pack launches an attack on it. The entire hunting process of the wolf pack is divided into concentrated attacks and dispersed attacks, corresponding to the local search and global search processes of the GWO algorithm, respectively. When the coefficient vector |A|≤1, the wolf pack launches the concentrated attack, and when |*A*| > 1, the wolf pack launches the dispersed attack.

### 2.2 Improved grey wolf optimization algorithm

#### 2.2.1 Improved Tent chaotic mapping initializing population.

The GWO algorithm uses the random method to generate initial positions during the population initialization stage. However, this may cause the initial grey wolf individuals to cluster in the small range, leading to problems such as premature algorithm convergence. The initial population’s quality will affect the positioning algorithm’s convergence speed and the optimal solution quality [26]. To make the population more evenly distributed in the search space, this paper uses Tent chaotic mapping to generate the initial positions of the population. The expression of the Tent chaotic mapping is as follows [27]:


$$\begin{array}{c} T(x)=\left\{ \begin{array}{c} \frac{x}{\upsilon },0<x<\upsilon \end{array}\right. \end{array}$$ (30)

Due to the characteristics of Tent chaotic sequences, short and unstable periodic points are often prone to occur. This paper sets a variable factor ϑ in the Tent chaotic mapping expression [19], and the improved Tent chaotic mapping expression is as follows:


$$\begin{array}{c} T^{\prime }(x)=\left\{ \begin{array}{c} \frac{x}{\upsilon }+\vartheta ,0<x<\upsilon \end{array}\right. \end{array}$$ (31)

where $\vartheta =rand\times \frac{1}{t_{\max}}$ and *rand* is a random number between 0 and 1.

The initial positions of the grey wolf individuals for Tent chaotic mapping are as follows:


$$\begin{array}{c} x_{0}=x_{lb}+(x_{ub}-x_{lb})T^{\prime }(x) \end{array}$$ (32)

where $x_{lb}$ is the upper bound of the grey wolf search area, and $x_{ub}$ is the lower bound of the grey wolf search area.

#### 2.2.2 Nonlinear convergence factor.

To balance the global and local search capabilities of the GWO algorithm, it is necessary to ensure a large convergence factor value and slow reduction speed in the early stages of iteration, maintain a large search range, and enhance global search capabilities. In the late stage of iteration, it is necessary to ensure that the convergence factor has a small value and decreases quickly to enhance local search capability [28]. Therefore, this paper designs a nonlinear convergence factor based on the cosine function to balance the algorithm’s global search and local exploitation abilities. The formula for the nonlinear convergence factor designed in this paper is as follows:


$$\begin{array}{c} a=\frac{1}{2}(a_{ini}-a_{fin})\times (1+\cos((\frac{t}{t_{max}}{)}^{\varphi }\times \pi )) \end{array}$$ (33)

where $a_{ini}$ is the initial value, $a_{fin}$ is the final value, and φ is the adjustment parameter.

#### 2.2.3 Dynamic sinusoidal inertia weight.

In the traditional GWO algorithm, the α,β, and δ wolves play the role of the decision-making layer of the population, guiding the whole wolf pack towards the target. However, although the GWO algorithm can quickly converge in the early stage, it is prone to get stuck in the local optimum in the later stage, resulting in insufficient search accuracy. To address this issue and improve the optimization ability of the GWO algorithm in different iterations, an inertia weight factor is introduced to improve the position update strategy of the GWO algorithm. Unlike traditional inertia weight adjustment methods such as TIGWO (Traditional Improved GWO), which often employ static or simple linearly decreasing weight strategies, the proposed MSGWO algorithm adopts a dynamic nonlinear inertia weight mechanism. The TIGWO approach typically uses fixed weight reduction patterns that cannot adequately respond to the algorithm’s real-time search state, while MSGWO’s dynamic weight adjusts according to the current iteration progress, better balancing global exploration and local exploitation.

The improved position update formulas are as follows:


$$\begin{array}{c} w=w_{ini}-(w_{ini}-w_{fin})\times \sin(\frac{\pi }{2}\times \frac{t}{t_{max}}) \end{array}$$ (34)

$$\begin{array}{c} \left\{ \begin{array}{c} X_{1}(t+1)=wX_{\alpha }(t)-A_{1}\times D_{\alpha }\\ X_{2}(t+1)=wX_{\beta }(t)-A_{2}\times D_{\beta } \end{array}\right. \end{array}$$ (35)

$$\begin{array}{c} X(t+1)=\frac{{\tilde{X}}_{1}(t+1)+{\tilde{X}}_{2}(t+1)+{\tilde{X}}_{3}(t+1)}{3} \end{array}$$ (36)

where $w_{ini}=0.95$, $w_{fin}=0.4$.

The sinusoidal adjustment of the inertia weight factor in Eq 34 enables a smooth transition from global exploration to local exploitation. In the early iterations, the weight remains relatively high, promoting extensive global search. As iterations progress, the weight gradually decreases in a nonlinear fashion, enhancing local refinement in later stages. This dynamic adaptation contrasts with TIGWO’s static or linear weight reduction, which often leads to premature convergence or insufficient local search precision. The proposed dynamic mechanism allows MSGWO to maintain better balance between exploration and exploitation throughout the optimization process.

### 2.3 Implementation of indoor positioning method with improved grey wolf optimization

#### 2.3.1 Determination of the search area.

The search area has a significant impact on the performance and efficiency of the GWO algorithm. The too-large search area can slow down the convergence speed of the algorithm. At the same time, the too-small search area may result in the optimum solution not being within the search area, thus affecting the accuracy of the algorithm. In the field of indoor positioning, the beneficial search area is the area near the positioning point. Suppose the estimated position of the positioning point can be obtained through preliminary positioning, and the search area of the GWO algorithm can be determined based on this position. In that case, it will inevitably improve the convergence speed of the algorithm and avoid the waste of computing resources [29].

**Fig 3:**
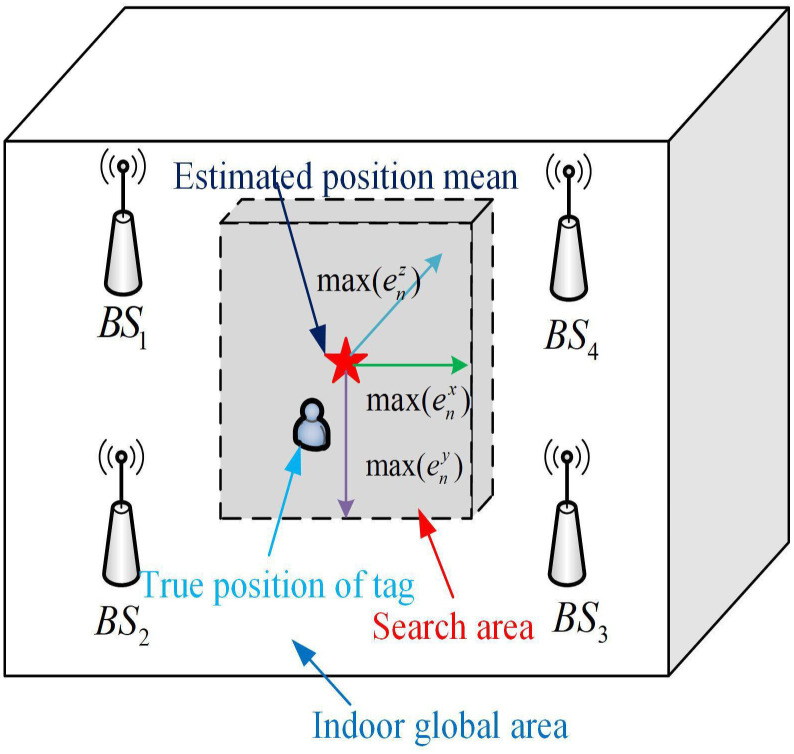
Determination of the search area. The search area is centered at the mean of Chan algorithm initial positioning results, with boundaries defined by the maximum positioning errors ($\max(e_{n}^{x})$, $\max(e_{n}^{y})$, $\max(e_{n}^{z})$) in each axis. This region, bounded by the four base stations ($BS_{1}--BS_{4}$) in the indoor environment, contains the tag’s true position and reduces unnecessary global searches for the subsequent MSGWO algorithm, lowering computational complexity.

The specific steps to determine the search area are as follows:

1) Collect *n* sets of TDOA measurements of tag position, solve *n* sets of positioning results using the Chan algorithm, and calculate the positioning error between the positioning results and the true position.
2) The mean of the positioning results is used as the center of the search area for the GWO algorithm. The mean of the positioning results and the range of error determine the initial search area of the GWO algorithm. The determination of the search area is shown in Fig 3. The specific formula representation is as follows:

The true position of the tag is (*x*, *y*, *z*); the Chan algorithm solves *n* sets of positioning results are $\left(x_{n},y_{n},z_{n}\right)$; the mean of the positioning results is $\left(\overset{―}{x},\overset{―}{y},\overset{―}{z}\right)$; the errors of position are $\left(e_{n}^{x},e_{n}^{y},e_{n}^{z}\right)$.


$$\begin{array}{c} \left\{ \begin{array}{c} e_{n}^{x}=\left|x_{n}-x\right|\\ e_{n}^{y}=\left|y_{n}-y\right| \end{array}\right. \end{array}$$ (37)

The population search area is as follows:


$$\begin{array}{c} \left[\begin{array}{ccc} \overset{―}{x}\pm \max(e_{n}^{x}) & \overset{―}{y}\pm \max(e_{n}^{y}) & \overset{―}{z}\pm \max(e_{n}^{z}) \end{array}\right] \end{array}$$ (38)

The maximum positioning errors along the X, Y, and Z axes are obtained through an offline calibration procedure. Specifically, a set of calibration points with known coordinates is first selected in the deployment area. The Chan algorithm is then repeatedly applied to estimate the positions of these calibration points, and the positioning errors along each axis are statistically analyzed. The maximum error value of each axis is finally adopted to determine the search region boundaries for subsequent localization tasks.

#### 2.3.2 Reconstruction of TDOA-based ranging measurements.

**Fig 4:**
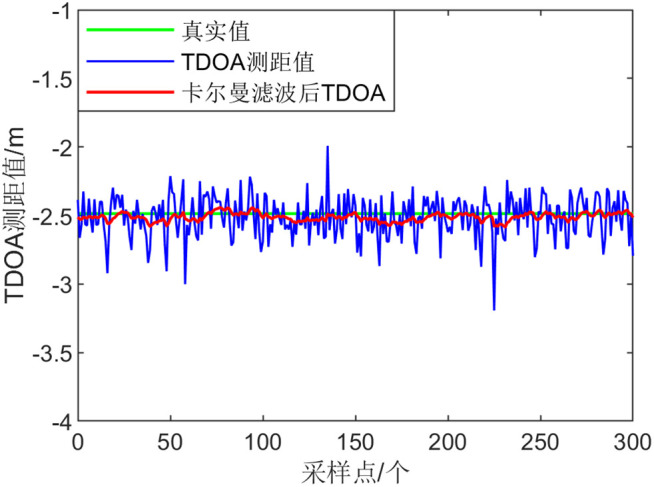
Kalman filter processing of TDOA ranging values. Comparison of true range, raw TDOA measurements contaminated by multipath and NLOS effects, and Kalman-filtered results. The filter effectively suppresses noise and improves ranging accuracy.

To mitigate the influence of partial multipath effects and NLOS errors, this paper employs the Kalman filter algorithm to correct the TDOA ranging measurements. The Kalman filter algorithm [71] can effectively utilize both current and historical observations to reconstruct the TDOA ranging value of the tag’s current state, thereby making the filtered TDOA ranging value approximate the true value. The simulation of TDOA ranging processed by the Kalman filter is illustrated in Fig 4.

#### 2.3.3 Objective function design.

In the TDOA positioning system, the only prior knowledge available is the TDOA measurement values received through communication between base stations and tags, as well as the coordinates of each base station. Therefore, the objective function can only be established based on these two pieces of information. In practical applications, TDOA measurement values are subject to noise, and this noise can vary with changes in the environment. To reduce the impact of noise on the positioning result, this paper adopts the Kalman filter algorithm [30,31] to process the TDOA measurement values. Therefore, the objective function designed in this paper seeks the optimal position based on the consistency between the distance difference calculated from the grey wolf individual position and the distance difference processed by Kalman filter. The smaller the value of the objective function, the better the positioning accuracy.

The positions of the base stations are $\left(x_{j},y_{j},z_{j}\right)$, the true position of the tag is (*x*, *y*, *z*), and the positions of the individual grey wolves in the population are $\left({\hat{x}}_{i},{\hat{y}}_{i},{\hat{z}}_{i}\right)$. The objective function is as follows:


$$\begin{array}{c} f_{i}=\frac{1}{m-1}\sum_{j=2}^{m}[\begin{array}{c} {\hat{r}}_{j,1}-(\sqrt{({\hat{x}}_{i}-x_{j}{)}^{2}+({\hat{y}}_{i}-y_{j}{)}^{2}+({\hat{z}}_{i}-z_{j}{)}^{2}}\\ -\sqrt{({\hat{x}}_{i}-x_{1}{)}^{2}+({\hat{y}}_{i}-y_{1}{)}^{2}+({\hat{z}}_{i}-z_{1}{)}^{2}}) \end{array}{]}^{2} \end{array}$$ (39)

where ${\hat{r}}_{j,1}$ is the TDOA measurement values after Kalman filter processing; *m* is the number of base stations.

#### 2.3.4 Algorithm steps.

The specific implementation steps of the indoor positioning method using MSGWO are as follows:

1) Perform initial positioning of the tag. The base stations communicate with the tag to collect TDOA measurement values. To suppress multipath effects and NLOS errors, the Kalman filter is employed to correct the raw TDOA measurements. The filtered TDOA values are substituted into the Chan algorithm to calculate the initial position of the tag.
2) Determine the search region for the population. The Chan algorithm solves for the initial position of the tag, constraining the population’s search region around the initial position.
3) Within the set population search area, initialize individual grey wolf populations through an improved Tent mapping and calculate individual fitness based on the objective function established by Eq 39.
4) The grey wolf individuals were ranked according to fitness value, and the top three individuals were α,β, and δ wolf.
5) Update the coefficient vectors *A* and *C* based on the nonlinear convergence factor of Eq 33.
6) According to Eqs 34, 35, and 36 update the position of the current grey wolf *w* using the dynamic inertia coefficient weight factor position update strategy. Recalculate the fitness of individuals and update the position information of the α,β, and δ wolves.
7) When the grey wolf population finds the global optimum or reaches the maximum number of iterations, the algorithm ends and outputs the optimal individual position.

**Fig 5:**
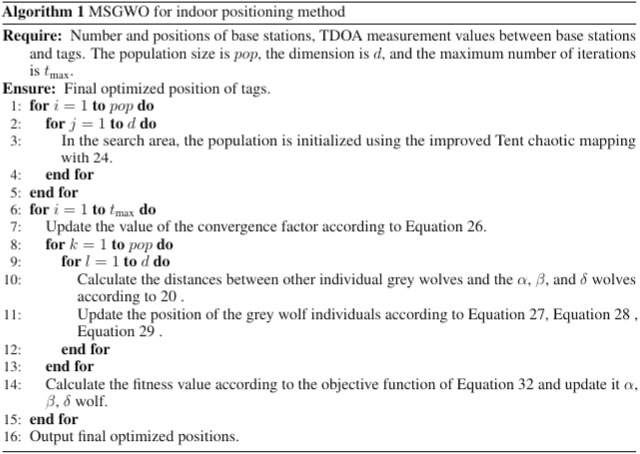
Pseudocode of the proposed MSGWO indoor positioning method. Including TDOA preprocessing (Kalman filter), initial position estimation (Chan algorithm), and the main MSGWO optimization loop. The loop updates the nonlinear convergence factor, calculates distances to the α, β, and δ wolves, updates positions via the inertia weight strategy, and updates the wolf hierarchy based on fitness.

The pseudocode of the proposed MSGWO algorithm is shown in Fig 5

#### 2.3.5 Complexity analysis.

The time complexity of GWO algorithm and the improved grey wolf algorithm (MSGWO) in this paper is analyzed below. Determine the search region for the population is determined by the initial position calculated using the Chan algorithm, the time complexity of Chan algorithm is $O(mn^{2}+n^{3})$, *m* is the amount of data in TDOA and *n* is the localization dimension. Let the number of population individuals be NP, the individual dimension be *d*, and the number of iterations be *t*. In the MSGWO algorithm, the *t*ime for Tent mapping to initialize the population is O(NP×d); the time for calculating individual fitness and selecting the three optimal individuals α,β, and δ wolves is *O*(*NP*); the time for updating the position of grey wolf individuals is O(t×NP×d). Since the Chan algorithm does not involve iteration, determination of the search area would be far less time costing than the search process, so the total time complexity of MSGWO algorithm is:


$$\begin{array}{c} O_{MSGWO}=O(mn^{2}+n^{3}+NP\times d+NP+t\times NP\times d)\approx O(t\times NP\times d) \end{array}$$ (40)

The GWO algorithm time complexity is also O(t×NP×d) [32]. In summary, the MSGWO algorithm does not add additional time costs and has the same time complexity as the GWO algorithm.

### 2.4 Experimental environment

**Fig 6:**
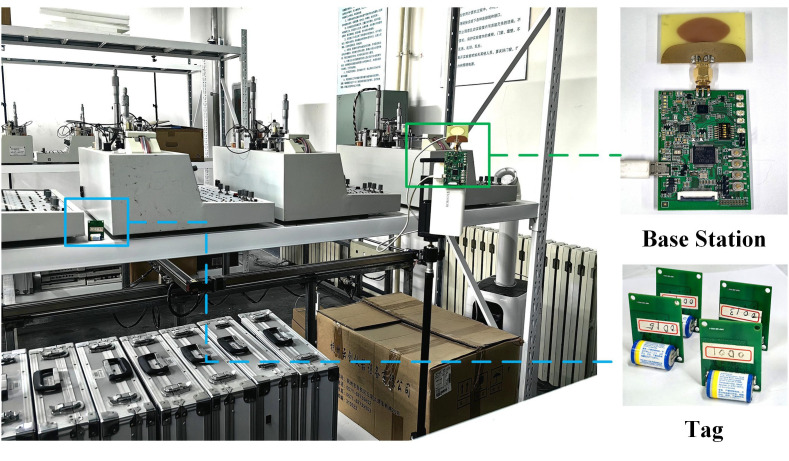
Actual equipment warehouse used for UWB indoor positioning experiments. The warehouse has a size of 8.3 m × 7.3 m × 3.7 m, with no obvious obstacles except for stacked equipment (consistent with the experimental scenario described in Materials and methods).

**Fig 7:**
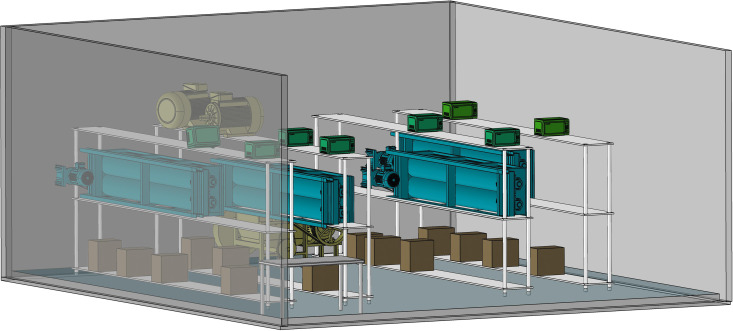
Simulated equipment warehouse scene for positioning algorithm verification. The scene is constructed based on the size of the actual warehouse (8.3 m × 7.3 m × 3.7 m), and the distribution of virtual obstacles is consistent with the actual warehouse to ensure simulation authenticity.

**Fig 8:**
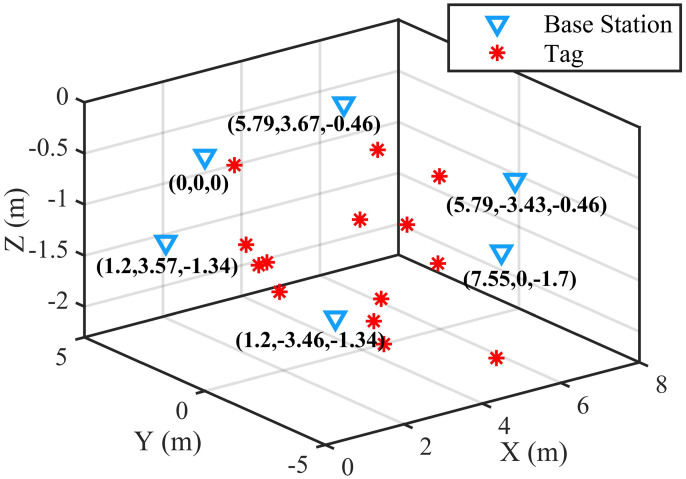
Deployment positions of base stations and tags in the actual warehouse. A total of 6 base stations are deployed (e.g., base station 1 at coordinate (0, 0, 0)), and 14 tag test points are evenly distributed in the warehouse (used for collecting TDOA measurement data and verifying positioning accuracy).

The indoor environment is shown in Fig 6. The indoor space measures 8.3 m × 7.3 m × 3.7 m, and 6 base stations and 14 tags were used for positioning. The simulated indoor equipment warehouse is shown in Fig 7. The positions of the base stations and tags are shown in Fig 8. The UWB devices and laser rangefinder are listed in Table 1.

**Table 1:** Experimental devices used in UWB indoor positioning experiments.

Devices	Function
UWB EB1003 chip	Used for tag positioning
STM32F105 MCU	Used to control the operation of the UWB chip
Laser rangefinder	Used to measure the true position of the tag

All devices are calibrated before experiments to ensure measurement accuracy; the laser rangefinder has a measurement precision of ±1 mm.

## 3 Results

### 3.1 Benchmark function testing and analysis

**Fig 9:**
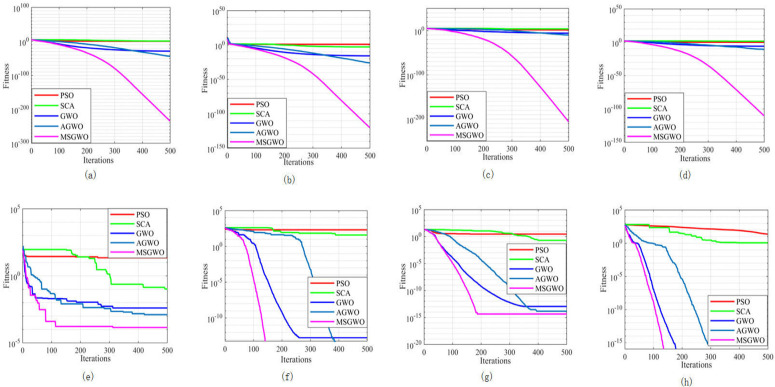
Convergence curves of $f_{1}--f_{8}$ test function. (a) *f*_1_ function; (b) *f*_2_ function; (c) *f*_3_ function; (d) *f*_4_ function; (e) *f*_5_ function; (f) *f*_6_ function; (g) *f*_7_ function; (h) *f*_8_ function.

This paper selected eight benchmark functions for testing [33] to validate the performance improvement of the GWO algorithm with Tent chaotic mapping, nonlinear convergence factor, and dynamic inertia weight factor. Table 2 shows the functions, search ranges, and dimensions for $f_{1}\sim f_{8}$ functions. Perform benchmark function testing experiments on PSO [34], SCA [35], GWO [36], AGWO [37], and the proposed MSGWO algorithm in this paper. Fig 9 shows the convergence curve of the $f_{1}\sim f_{8}$ test function. Table 3 shows the benchmark function test results.

**Table 2:** Benchmark test functions.

Function	Range	*f*_min_	Type
$f_{1}=\sum_{i=1}^{n}x_{i}^{2}$	[−100,100]	0	Unimodal
$f_{2}=\sum_{i=1}^{n}|x_{i}|+\prod_{i=1}^{n}|x_{i}|$	[−10,10]	0	Unimodal
$f_{3}=\sum_{i=1}^{n}{\left(\sum_{j=1}^{i}x_{j}\right)}^{2}$	[−100,100]	0	Unimodal
$f_{4}=\max_{i}\{|x_{i}|,1\leq i\leq n\}$	[−100,100]	0	Unimodal
$f_{5}=\sum_{i=1}^{n}\left(x_{i}^{2}+\text{rand}(0,1)\right)$	[−128,128]	0	Unimodal
$f_{6}=\sum_{i=1}^{n}\left(x_{i}^{2}-10\cos(2\pi x_{i})+10\right)$	[−5.12,5.12]	0	Multimodal
$f_{7}=-20\exp\left(-0.2\sqrt{\frac{1}{n}\sum_{i=1}^{n}x_{i}^{2}}\right)-\exp\left(\frac{1}{n}\sum_{i=1}^{n}\cos(2\pi x_{i})\right)+20+e$	[−32,32]	0	Multimodal
$f_{8}=\frac{1}{4000}\sum_{i=1}^{n}x_{i}^{2}-\prod_{i=1}^{n}\cos\left(\frac{x_{i}}{\sqrt{i}}\right)+1$	[−600,600]	0	Multimodal

All tests use population size = 30, maximum iterations = 500, dimension *d* = 30; each algorithm runs 30 times independently via Monte Carlo method to calculate average (Ave) and standard deviation (Std).

**Table 3:** Benchmark function test results.

Function	Value	PSO	SCA	GWO	AGWO	MSGWO
*f*_1_	Ave	3.91E + 00	1.40E + 01	9.91E-28	4.06E-43	3.30E-235
*f*_1_	Std	5.54E-01	2.40E + 01	1.31E-27	1.20E-42	2.45E-230
*f*_2_	Ave	8.44E + 00	2.32E-02	1.15E-16	5.80E-27	2.50E-120
*f*_2_	Std	5.82E-01	2.75E-02	8.07E-17	6.05E-27	2.87E-120
*f*_3_	Ave	4.06E + 01	9.48E + 03	6.33E-06	5.59E-10	4.31E-206
*f*_3_	Std	1.12E + 01	5.80E + 03	1.94E-05	1.66E-09	3.11E-194
*f*_4_	Ave	8.79E-01	3.40E + 01	6.29E-07	1.44E-11	1.23E-110
*f*_4_	Std	9.01E-02	1.43E + 01	5.14E-07	2.49E-11	1.63E-110
*f*_5_	Ave	1.78E + 01	1.30E-01	1.60E-03	1.34E-03	1.02E-04
*f*_5_	Std	4.01E + 00	1.15E-01	1.27E-03	7.89E-04	9.36E-05
*f*_6_	Ave	2.02E + 02	5.24E + 01	2.85E + 00	1.40E-04	0.00E + 00
*f*_6_	Std	1.32E + 01	5.21E + 00	4.23E + 00	7.66E-04	1.47E-196
*f*_7_	Ave	3.00E + 00	1.71E + 01	1.04E-13	8.59E-15	4.44E-15
*f*_7_	Std	1.37E-01	7.21E + 00	1.55E-14	1.89E-15	1.09E-150
*f*_8_	Ave	2.68E + 01	1.01E + 00	4.48E-03	9.26E-04	0.00E + 00
*f*_8_	Std	6.54E + 00	3.63E-01	8.42E-03	5.07E-03	1.32E-100

All tests use population size = 30, maximum iterations = 500, dimension *d* = 30; each algorithm runs 30 times independently via Monte Carlo method. “Ave” = average optimization value, “Std” = standard deviation; smaller Ave and Std indicate better algorithm performance and stability.

In the experiment, PSO, SCA, GWO, AGWO, and MSGWO algorithms all adopt the same settings: the population size is 30, the number of iterations is 500, and the dimension *d* is 30. The Monte Carlo method was used to run the test function 30 times independently, and the average (Ave) and standard deviation (Std) obtained for each algorithm in the 30 runs are recorded. The smaller Ave value indicates better algorithm performance, while the smaller Std value indicates better algorithm stability [38]. From the convergence curves of the $f_{1}\sim f_{8}$ test functions in Fig 9, MSGWO algorithm improves the population diversity and enhances the convergence speed.

According to the benchmark test results in Table 3, the MSGWO algorithm outperforms the PSO, SCA, GWO, and AGWO algorithms, as indicated by its smaller average (Ave) and standard deviation (Std) values across the *f*_1_ through *f*_8_ functions. This highlights the superior optimization accuracy and stability of the MSGWO algorithm.

### 3.2 Simulation positioning experiment

To verify the performance of the proposed MSGWO algorithm in indoor positioning, simulation experiments were conducted. The positions of the base stations in the simulation experiment are the same as those in the actual indoor experiment. The positions of the base stations are *BS*_1_ (0, 0, 0), $BS_{2}(1.2,3.57,-1.34)$, $BS_{3}(5.79,3.67,-0.46)$, $BS_{4}(7.55,0,-1.7)$, $BS_{5}(5.79,-3.43,-0.46)$, and $BS_{6}(1.2,-3.46,-1.34)$. The tag position is MS(5.2,1.3,−0.6). The simulated experiment noise follows a Gaussian distribution with zero mean and standard deviation σ.

To verify the positioning performance of the algorithm, root mean square error (RMSE) [39], absolute error in the *X*-axis, absolute error in the *Y*-axis, absolute error in the *Z*-axis, and cumulative distribution function (CDF) are used as evaluation metrics for positioning accuracy. The RMSE represents the square root of the squared sum of the mean values of the differences between the true and estimated positions. The lower the value of the RMSE, the higher the positioning accuracy. CDF can be used to measure the cumulative probability that the positioning error between the estimated position and the real position is less than or equal to the threshold value. The specific formulas are as follows:


$$\begin{array}{c} RMSE=\sqrt{\frac{1}{N}\sum_{r=1}^{N}\left[{\left(x_{r}^{\prime }-x\right)}^{2}+{\left(y_{r}^{\prime }-y\right)}^{2}+{\left(z_{r}^{\prime }-z\right)}^{2}\right]} \end{array}$$ (41)

$$\begin{array}{c} \Delta x=\left|x_{r}^{\prime }-x\right| \end{array}$$ (42)

$$\begin{array}{c} \Delta y=|\begin{array}{c} y_{r}^{\prime }-y \end{array}| \end{array}$$ (43)

$$\begin{array}{c} \Delta z=|z_{r}^{\prime }-z| \end{array}$$ (44)

$$\begin{array}{c} CDF(\mu )=P(e\leq \mu ) \end{array}$$ (45)

where *N* represents the number of experiments, $\left(x_{r}^{\prime },y_{r}^{\prime },z_{r}^{\prime }\right)$ represents the estimated positions of the tags, and (*x*, *y*, *z*) represents the true position of the tag.

#### 3.2.1 Simulation convergence performance analysis.

**Fig 10:**
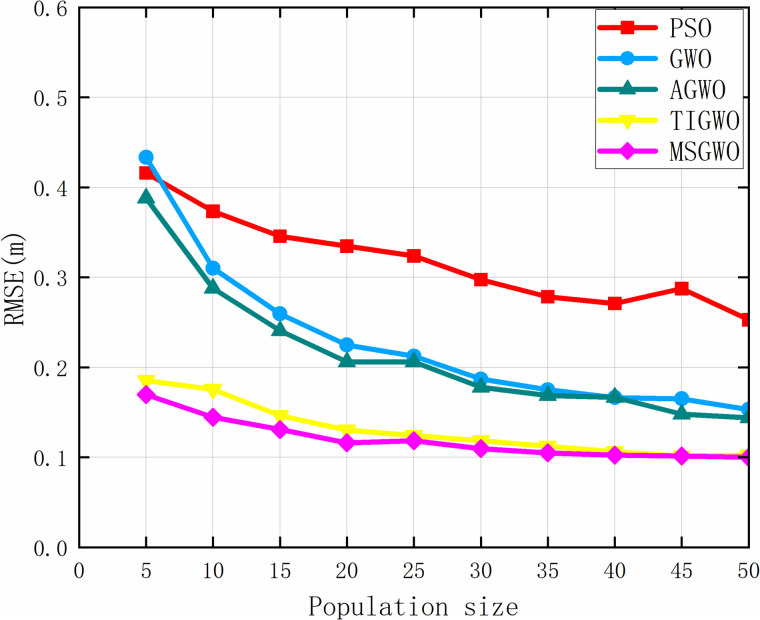
Influence of the population size on positioning accuracy. Simulation results showing the effect of population size (10–50) on positioning accuracy for the MSGWO algorithm in a virtual indoor warehouse (8.3 m × 7.3 m × 3.7 **m)**.

To determine the population size required for the algorithm to run, experiments were conducted under the condition of a ranging noise standard deviation σ of 0.3 m. The influence of the population size on the positioning error RMSE value was observed, as shown in Fig 10.

From Fig 10, the positioning error of PSO, GWO, AGWO, and MSGWO algorithms remains stable when the population size is around 30. Further increasing the population size does not have a significant effect on improving the positioning accuracy, and it also increases the running time of the algorithm. Therefore, considering both the running time and positioning accuracy, the population size of 30 is set for the experiment.

The PSO, GWO, AGWO, and MSGWO algorithms were subjected to 300 localization experiments at different population sizes, and the algorithm running time is shown in Table 4. Considering the guarantee of positioning accuracy while reducing time consumption, so this paper chooses to conduct positioning experiments when the number of populations is 30.

**Table 4:** Comparison of Algorithm Runtime for Different Population Sizes (Unit: s).

Population Size	PSO	GWO	AGWO	TIGWO	MSGWO
10	3.312	3.174	3.150	3.804	2.971
15	4.805	4.756	5.372	4.423	4.395
20	6.323	6.273	7.011	6.092	5.756
25	8.019	7.508	7.669	7.223	6.973
30	9.391	9.053	9.264	8.776	8.499
35	11.077	11.171	10.503	10.345	10.282
40	12.289	11.943	12.015	11.402	11.190
45	14.972	14.790	13.986	13.468	12.801
50	15.363	15.328	14.529	14.492	14.341

All runtime data are obtained from 300 localization experiments; MSGWO has the shortest runtime due to its constrained search area (reducing computational resource waste). Population size = 30 is selected for subsequent experiments (balancing runtime and positioning accuracy).

**Fig 11:**
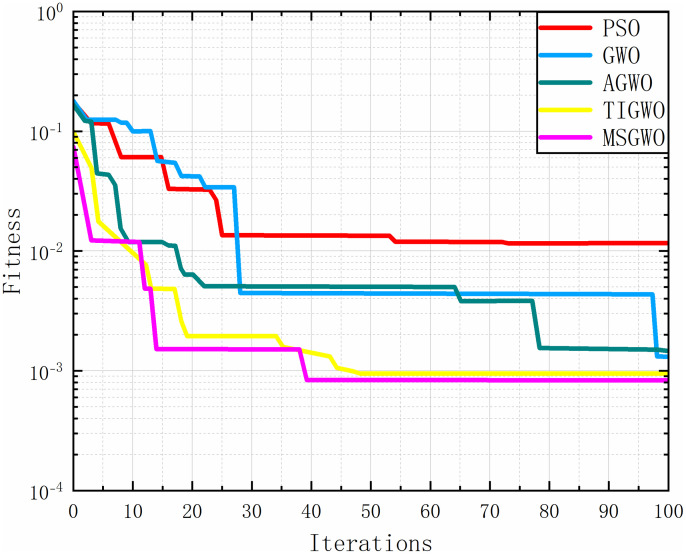
Relationship between fitness value and the number of iterations. Simulation results showing the relationship between fitness value and iteration count (1–100) for PSO, GWO, AGWO, TIGWO, and MSGWO algorithms in 3D UWB indoor positioning; population size = 30, with fitness value calculated via the TDOA-based objective function.

**Fig 12:**
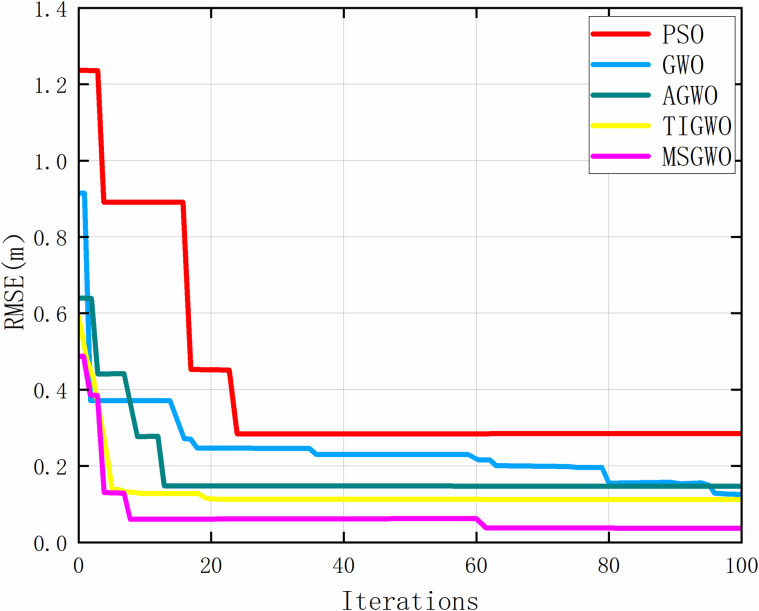
Relationship between RMSE value and the number of iterations. Simulation results illustrating the relationship between positioning error (RMSE) and iteration count (1–100) for PSO, GWO, AGWO, TIGWO, and MSGWO algorithms; tested in a virtual indoor warehouse, with RMSE derived from 300 independent positioning trials.

To verify the convergence performance of the MSGWO algorithm, experiments were conducted under the condition that the number of populations was 30 and the number of iterations was 100, Fig 11 shows the relationship between fitness value and the number of iterations, and Fig 12 shows the relationship between RMSE value and the number of iterations.

From Fig 11, it can be observed that in the early stage of the algorithm, as the number of iterations increases, the fitness value shows a gradually decreasing trend. In the later stage, the fitness value tends to stabilize. Compared with the MSGWO algorithm, PSO, GWO, and AGWO algorithms have slower convergence speeds. From Fig 12, it can be observed that with the increase in the number of iterations, the positioning error RMSE value of the PSO, GWO, AGWO, and MSGWO algorithms gradually decreases and eventually stabilizes. Remarkably, the MSGWO algorithm demonstrates a faster convergence rate and superior positioning accuracy compared to the other algorithms.

#### 3.2.2 Simulation positioning accuracy analysis.

To verify the positioning performance of the proposed algorithm, 300 Monte Carlo simulation experiments were conducted on the PSO, GWO, AGWO and MSGWO algorithms under different ranging error standard deviation σ. The experimental results are shown in Table 5.

**Table 5:** Comparison of RMSE Values under Different Ranging Error Standard Deviations (Unit: m).

σ	PSO	GWO	AGWO	TIGWO	MSGWO
0.1	0.234	0.175	0.147	0.085	0.081
0.2	0.299	0.175	0.158	0.123	0.099
0.3	0.325	0.185	0.179	0.143	0.109
0.4	0.339	0.204	0.205	0.186	0.141
0.5	0.351	0.259	0.224	0.207	0.165

This table presents the root mean square error (RMSE) of positioning results for PSO, GWO, AGWO, and MSGWO algorithms under different ranging error standard deviations (σ: 0.1–0.5 m). Data are derived from 300 Monte Carlo simulation experiments in a virtual 3D UWB indoor positioning scenario, with σ representing the Gaussian noise level of TDOA measurements.

From Table 5, it can be observed that as the standard deviation σ of the ranging error increases, the positioning error RMSE value of the PSO, GWO, AGWO, and MSGWO algorithms also increases. Compared with the PSO, GWO, and AGWO algorithms, the proposed MSGWO algorithm has a smaller positioning error and can achieve better positioning performance.

### 3.3 Actual positioning experiment

#### 3.3.1 Actual positioning accuracy analysis.

In the equipment warehouse, 300 positioning tests were conducted on 14 tag positions using the MSGWO algorithm proposed in this paper. The average positioning result is the final optimized position of the tag. The experimental results are shown in Table 6.

**Table 6:** Experimental data (Unit: m).

No.	True position	Optimization position	Δ𝐱	Δ𝐲	Δ𝐳
1	(2.35,2.60,−0.60)	(2.24,2.33,−0.67)	0.11	0.27	0.07
2	(5.20,1.30,−0.60)	(5.08,1.14,−0.79)	0.12	0.16	0.19
3	(5.24,−1.20,−0.60)	(5.12,−1.32,−0.57)	0.12	0.12	0.03
4	(2.41,2.51,−0.60)	(2.44,2.50,−0.99)	0.03	0.01	0.39
5	(5.20,−1.20,−1.45)	(5.21,−1.13,−1.62)	0.01	0.07	0.17
6	(2.50,1.50,−1.45)	(2.56,1.57,−1.46)	0.06	0.07	0.01
7	(2.80,2.85,−1.45)	(2.52,2.70,−1.18)	0.28	0.15	0.27
8	(6.10,1.55,−1.45)	(6.16,1.52,−1.50)	0.06	0.03	0.05
9	(2.26,1.45,−1.45)	(2.06,1.65,−1.62)	0.20	0.20	0.17
10	(4.30,0,−2.05)	(4.09,0.02,−2.01)	0.21	0.02	0.04
11	(3.27,−2.10,−1.95)	(3.07,−2.17,−2.06)	0.20	0.07	0.11
12	(3.40,2.44,−1.93)	(3.16,2.43,−2.11)	0.24	0.01	0.18
13	(5.86,−2.55,−2.30)	(5.94,−2.66,−2.28)	0.08	0.01	0.02
14	(6.14,2.69,−2.30)	(6.15,2.65,−2.40)	0.01	0.04	0.10

The data is obtained from 300 positioning experiments on 14 tags in an 8.3 m × 7.3 m × 3.7 m indoor equipment warehouse; “Optimization position” refers to the tag position optimized by the MSGWO algorithm, and Δx/Δy/Δz represent the absolute positioning errors in X/Y/Z axes respectively.

From Table 6, the MSGWO algorithm has a maximum absolute error of 0.28 m, a minimum of 0.01 m, and an average of 0.123 m in the *X*-axis. In the *Y*-axis, the maximum absolute error is 0.27 m, the minimum is 0.01 m, and the average is 0.088 m. In the *Z*-axis, the maximum absolute error is 0.39 m, the minimum is 0.01 m, and the average is 0.128 m. From the experimental data results, the MSGWO algorithm has high positioning accuracy in the *X*-axis, *Y*-axis, and *Z*-axis.

#### 3.3.2 Algorithm comparison.

**Fig 13:**
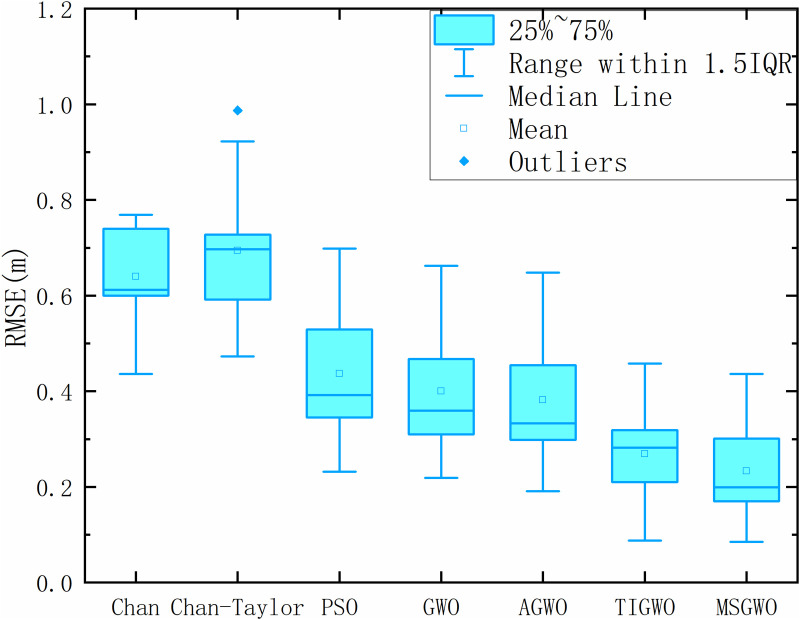
Box plot of positioning errors for different algorithms. The box plot statistically shows the positioning error distribution of six algorithms (Chan, Chan-Taylor, PSO, GWO, AGWO, TIGWO, MSGWO) across 14 tag positions in the actual indoor experiment.

In order to further verify the positioning performance of the proposed algorithm, this paper conducted comparative experiments using Chan [40], Chan-Taylor [41], PSO [34], GWO [36], AGWO [37], and the proposed MSGWO algorithm. The statistical results of positioning errors for 14 tag positions on different algorithms are shown in Table 7 and Fig 13.

**Table 7:** Statistical results of positioning error for different algorithms (Unit: m).

Algorithms	Maximum RMSE	Minimum RMSE	Average RMSE
Chan	0.769	0.436	0.631
Chan-Taylor	0.987	0.473	0.697
PSO	0.698	0.232	0.431
GWO	0.662	0.219	0.396
AGWO	0.648	0.191	0.376
TIGWO	0.392	0.073	0.255
MSGWO	0.436	0.085	0.234

The data is obtained from 300 positioning experiments on 14 tags in an indoor equipment warehouse (8.3 m × 7.3 m × 3.7 m). Compared with Chan, Chan-Taylor, PSO, GWO, and AGWO algorithms, the average positioning accuracy of MSGWO is improved by 62.92

From Table 7, the MSGWO algorithm has a lower maximum RMSE value, minimum RMSE value, and average RMSE value. Compared to the Chan, Chan-Taylor, PSO, GWO, and AGWO algorithms, the MSGWO algorithm improves the average positioning accuracy by 62.92%, 66.43%, 45.71%, 40.91%, and 37.76%, respectively.

Fig 13 shows the box plot of the positioning error statistical results for 14 tag positions. From Fig 13, the mean and median values of the positioning error for the MSGWO algorithm are lower than those of the Chan, Chan-Taylor, PSO, GWO, and AGWO algorithms. Therefore, the proposed MSGWO algorithm in this paper can achieve better positioning performance.

**Fig 14:**
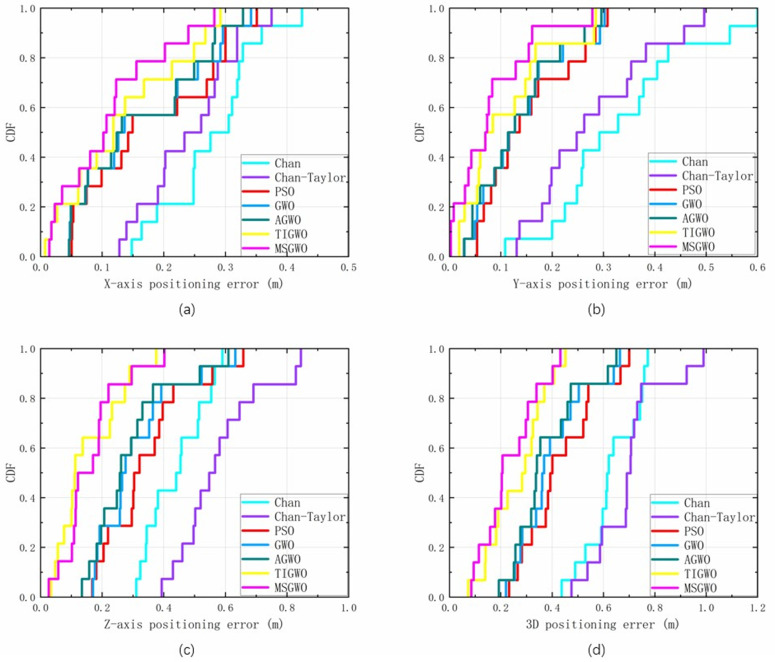
Positioning error CDF curves of different algorithms for each axis and 3D space. This figure shows the cumulative distribution function (CDF) curves of positioning errors for six algorithms (Chan, Chan-Taylor, PSO, GWO, AGWO, TIGWO, MSGWO) in the actual indoor experiment, covering four dimensions.

Fig 14 presents the cumulative distribution function (CDF) curves of positioning errors along the X-axis, Y-axis, Z-axis, and 3-D for the Chan, Chan-Taylor, PSO, GWO, AGWO, and MSGWO algorithms. As observed in Fig 14, the positioning errors at the 90th percentile on the X-axis for these algorithms are 0.359 m, 0.319 m, 0.300 m, 0.296 m, 0.283 m, and 0.240 m, respectively. On the Y-axis, the positioning errors at the 90th percentile are 0.546 m, 0.457 m, 0.284 m, 0.293 m, 0.263 m, and 0.161 m, respectively. For the Z-axis, the errors are 0.566 m, 0.829 m, 0.558 m, 0.523 m, 0.517 m, and 0.296 m, respectively. Finally, in the 3-D space, the positioning errors at the 90th percentile are 0.759 m, 0.924 m, 0.666 m, 0.640 m, 0.617 m, and 0.402 m, respectively. These results indicate that the proposed MSGWO algorithm consistently achieves higher positioning accuracy across all axes and in 3-D compared to the other algorithms. The significant reduction in positioning errors highlights the effectiveness of MSGWO in improving localization precision, making it a promising approach for high-accuracy positioning applications.

The average RMSE values for the 14 tag positions on the *X*-axis, *Y*-axis, and *Z*-axis are shown in Table 8. The average RMSE value for the *X*-axis is referred to as *X*-RMSE, the average RMSE value for the *Y*-axis is referred to as *Y*-RMSE, and the average RMSE value for the *Z*-axis is referred to as *Z*-RMSE. From Table 8, the proposed MSGWO algorithm has improved the average positioning accuracy on the *X*-axis by 59.57%, 53.33%, 36.36%, 31.29%, and 29.56% compared to the Chan, Chan-Taylor, PSO, GWO, and AGWO algorithms, respectively. On the *Y*-axis, the average positioning accuracy has been improved by 75.60%, 70.86%, 46.71%, 41.30%, and 39.10%, respectively. On the *Z*-axis, the average positioning accuracy has been improved by 63.01%, 71.92%, 52.21%, 47.23%, and 43.75%, respectively.

**Table 8:** Positioning errors of different algorithms on the X-axis, Y-axis, and Z-axis (Unit: m).

Algorithms	X-RMSE	Y-RMSE	Z-RMSE
Chan	0.277	0.332	0.438
Chan-Taylor	0.240	0.278	0.577
PSO	0.176	0.152	0.339
GWO	0.163	0.138	0.307
AGWO	0.159	0.133	0.288
TIGWO	0.129	0.102	0.153
MSGWO	0.112	0.081	0.162

The data is derived from 300 positioning experiments on 14 tags in an 8.3 m × 7.3 m × 3.7 m indoor equipment warehouse; *X*-RMSE/*Y*-RMSE/*Z*-RMSE represent the average root mean square error of positioning results in X/Y/Z axes respectively. Compared with Chan algorithm, MSGWO improves positioning accuracy by 59.57

#### 3.3.3 Statistical significance analysis.

To further validate the statistical significance of the performance improvements, this section conducts a non-parametric Wilcoxon signed-rank test based on the RMSE results from 14 test points and 300 independent runs. Pairwise comparisons are performed between the proposed MSGWO algorithm and the comparative algorithms (PSO, GWO, and AGWO), with the significance level set to α=0.01.

**Fig 15:**
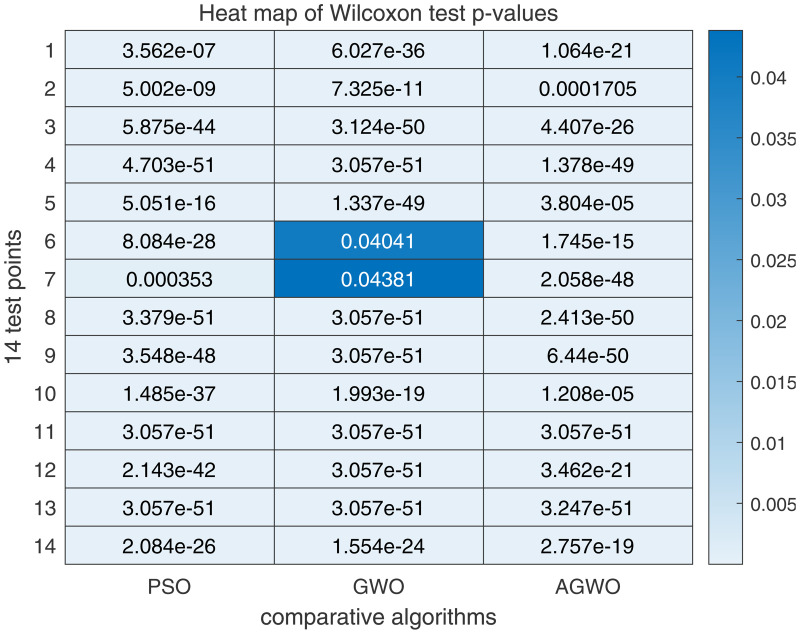
Heat map of Wilcoxon test p-values between MSGWO and comparative algorithms. Heat map of p-values obtained from the Wilcoxon signed-rank test for pairwise comparisons between MSGWO and other algorithms (PSO, GWO, and AGWO) across 14 test points. Most p-values are below 0.01, indicating statistically significant differences.

The distribution of p-values is illustrated in Fig. 15. In the heat map, lighter colors indicate smaller p-values and thus stronger statistical significance. It can be observed that the vast majority of p-values are significantly smaller than 0.01. Only two test points in the comparison with GWO yield p-values of 0.04041 and 0.04381, which still satisfy the significance criterion at the α=0.05 level.

These results demonstrate that the improvements in positioning accuracy achieved by MSGWO are not due to random variations, but represent statistically significant and consistent performance gains, thereby validating the effectiveness and reliability of the proposed algorithm.

## 4 Discussion

### 4.1 Parameter sensitivity analysis

**Fig 16:**
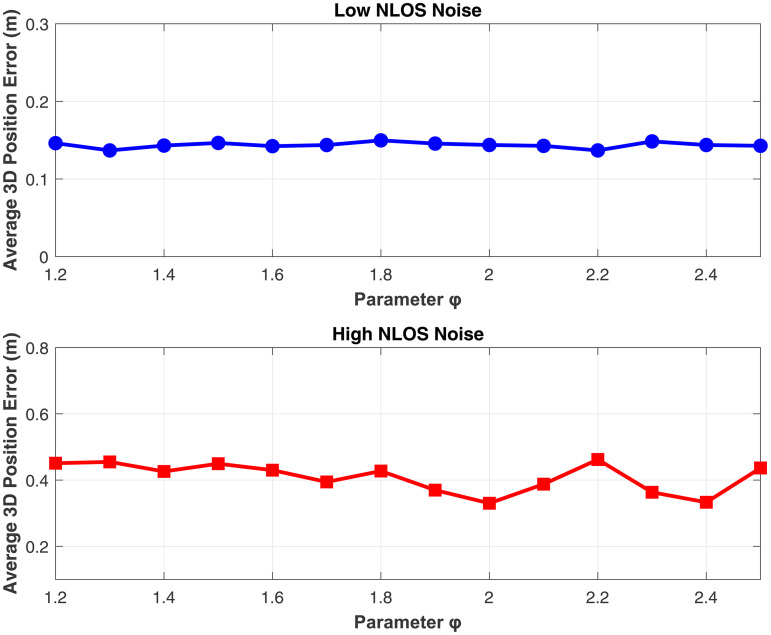
Parameter sensitivity analysis. Parameter sensitivity analysis of the nonlinear convergence factor φ. The average 3D positioning error is plotted versus φ in low-noise and high-noise scenarios, where φ∈[1.2,2.5]. The horizontal axis represents the parameter φ, and the vertical axis denotes the average positioning error **(m)**.

To analyze the effect of the nonlinear convergence factor parameter φ on the algorithm performance, experiments are conducted under two typical scenarios, i.e., low-noise and high-noise conditions. The parameter φ is varied within the range of [1.2, 2.5]. For each setting, the algorithm is independently executed multiple times, and the average 3D positioning error is recorded. The results are shown in Fig 16.

As observed from Fig 16, in the low-noise scenario, the positioning error remains stable at approximately 0.15 m, with only slight fluctuations as φ varies. In the high-noise scenario, although the overall error increases to the range of 0.3–0.5 m, the differences among different φ values are still relatively small.

Further analysis indicates that when φ lies within the interval of 1.8–2.2, the algorithm achieves a favorable balance between positioning accuracy and convergence stability. These results demonstrate that the proposed algorithm exhibits a certain degree of robustness with respect to φ within a reasonable range, maintaining stable performance under different noise conditions. This property reduces the difficulty of parameter tuning and enhances the practical applicability of the method.

### 4.2 Collaborative optimization analysis of the improved strategies

The three improvement strategies of the MSGWO algorithm are not isolated; instead, they address the core limitations of the traditional GWO algorithm in 3D UWB indoor positioning scenarios through “collaborative optimization,” a conclusion that can be cross-validated by benchmark function tests and positioning experiment results.

In the population initialization stage, the traditional GWO adopts a random generation method, which easily leads to the clustering of initial individuals and premature convergence. In contrast, the MSGWO introduces an improved Tent chaotic mapping with a variable factor ϑ. Changing the linear convergence factor to the nonlinear one balances the global and local optimization abilities of the algorithm. The dynamic inertia weight factor position updating strategy improves the optimization accuracy of the algorithm. The above improvement strategies enable the MSGWO algorithm to exhibit good optimization performance. From the unimodal functions *f*_1_ through *f*_5_, the MSGWO algorithm demonstrates a significantly faster convergence rate compared to the PSO, SCA, GWO, and AGWO algorithms. This suggests that the improved Tent chaotic mapping initialization strategy enhances both convergence speed and optimization accuracy. In the multimodal functions *f*_6_ through *f*_8_, the MSGWO algorithm requires fewer iterations and shows clear superiority in the convergence process, enabling it to quickly find the global optimum.

In terms of balancing regulation during the optimization process, the MSGWO replaces the traditional linear convergence factor (Eq. 19) with a nonlinear form based on the cosine function, achieving a dynamic balance where a large a value enhances global search in the early iteration stage and a small *a* value focuses on local optimization in the later stage. This strategy shows obvious advantages in the multimodal functions *f*_6_ to *f*_8_: PSO and SCA tend to fall into local optima (e.g., the Ave value of PSO in *f*_6_ is 2.02*E* + 02), while AGWO can avoid local optima but has insufficient accuracy. In contrast, the MSGWO not only converges to the theoretical optimal value (the Ave values of *f*_6_ and *f*_8_ are both 0) but also has a convergence speed 20% faster than that of AGWO. In addition, the MSGWO dynamically adjusts the inertia weight through a sine function, a large weight (0.95) is used in the early stage to enhance global optimization ability, and a small weight (0.4) is used in the later stage to improve local accuracy. This enables the optimization accuracy of the *f*_7_ function to reach 4.44E−15, further verifying the collaborative effectiveness of the improvement strategies.

From the results in Table 4, the more the number of populations, the more time consumed for the algorithm to run. The MSGWO algorithm has the shortest running time compared to the other algorithms. This is mainly since the MSGWO algorithm sets the search area of the populations, which avoids the waste of computational resources and reduces part of the time consumption. When the number of populations is less than or equal to 30, the positioning time of all four algorithms is less than 10s.The MSGWO algorithm uses an improved Tent chaotic mapping to initialize the population, which leads to the more uniform distribution of the population and thus enables faster convergence towards the vicinity of the optimum solution.

### 4.3 Analysis of research limitations

Despite the superior positioning performance achieved in this study, the proposed MSGWO algorithm still has several limitations in practical deployment, which are worth noting and improving in future work.First, the proposed method relies on an offline calibration procedure to determine the maximum positioning errors along the X-, Y-, and Z-axes. This process requires sufficient known calibration points and manual pre-processing, meaning the system cannot achieve fully plug-and-play deployment without prior knowledge.Second, the performance of the Kalman filter is sensitive to the selection of process noise covariance Q and measurement noise covariance R. These parameters need to be manually tuned according to the actual environment, and may require readjustment when the indoor environment changes significantly, indicating that the robustness can be further enhanced.Third, the search-region constraint of MSGWO depends heavily on the initial positioning accuracy of the Chan algorithm. In harsh environments with severe NLOS errors or complete obstruction, if the Chan algorithm produces large deviations or even fails, the preset search region will deviate from the real target position, thereby downgrading the positioning accuracy or causing failure of MSGWO.Fourth, the experiments are conducted in a fixed-scale indoor scenario (8.3 m × 7.3 m × 3.7 m). The performance of the proposed method in large spaces, dynamic moving tags, dense multi-target scenarios, or complex outdoor environments has not been verified.

## 5 Conclusion

In order to improve positioning accuracy in complex indoor environments, this paper proposes an MSGWO algorithm for 3D UWB indoor positioning. Firstly, the initial position of the tag is solved using the Chan algorithm, and the search area of the GWO algorithm is constructed with the initial position as the center. Then, the GWO algorithm is improved using the improved Tent chaotic mapping, nonlinear convergence factor, and dynamic inertia weight factor, and the MSGWO algorithm is proposed. Finally, within the set search area, the MSGWO algorithm is used to find the optimized position of the tag. The experimental results show that the proposed MSGWO algorithm can significantly improve indoor positioning accuracy and convergence speed. Compared with the Chan, Chan-Taylor, PSO, GWO, and AGWO algorithms, the MSGWO algorithm has an average positioning accuracy improvement of 62.92%, 66.43%, 45.71%, 40.91%, and 37.76% in 3-D overall, respectively, and has high practical application value.

## Supporting information

S1 Data Experimental datasets for the 14 test locations used to evaluate the positioning performance of the proposed grey wolf optimization algorithm. (ZIP)
